# Different Contributions of Physical Activity on Arterial Stiffness between Diabetics and Non-Diabetics

**DOI:** 10.1371/journal.pone.0160632

**Published:** 2016-08-10

**Authors:** Takeshi Iwasa, Eisuke Amiya, Jiro Ando, Masafumi Watanabe, Takahide Murasawa, Issei Komuro

**Affiliations:** 1 Department of Cardiovascular Medicine, Graduate School of Medicine, The University of Tokyo, Tokyo, Japan; 2 Department of Medical Engineering, The University of Tokyo, Tokyo, Japan; 3 Department of General Internal Medicine, Oncologic Emergencies, National Cancer Center Hospital, Tokyo, Japan; Osaka Shiritsu Daigaku, JAPAN

## Abstract

**Background:**

We compared the contribution of physical activity to the change in arterial stiffness between patients with and without diabetes in ischemic heart disease.

**Methods:**

We studied 96 (diabetes) and 109 (without diabetes) patients with ischemic heart disease treated by percutaneous coronary intervention (PCI). Arterial stiffness was assessed by cardio-ankle vascular index (CAVI) at the first diagnosis of significant coronary ischemia and 6 months after PCI and optimal medical therapy. Physical activity was evaluated using the long form of the International Physical Activity Questionnaire (IPAQ).

**Results:**

CAVI values increased more for diabetic patients than for non-diabetic. The IPAQ scores did not differ between the two groups. During follow-up, CAVI values did not significantly change in either group. In diabetic patients, the CAVI score for 48 patients did not change (NC-group) and 48 patients improved (Improved-group). Physical activity scores were 937.9 ± 923.2 and 1524.6 ± 1166.2 in the NC- and Improved-groups, respectively. IPAQ scores and uric acid levels significantly affect CAVI improvement after adjusting for age, sex, baseline CAVI, total cholesterol, and estimated glomerular filtration rate.

**Conclusion:**

Determining factors influencing CAVI improvement during follow-up were significantly different between patients with and without diabetes. IPAQ scores and uric acid levels were significantly correlated with CAVI changes.

## Introduction

Cardiovascular problems are the most important complications that critically determine the survival of patients with diabetes. The presence of diabetes intensely accelerates vascular remodeling, leading to an increase in arterial stiffness, and as a result, leads to the development of various atherosclerotic diseases such as ischemic heart disease or carotid stenosis [1]. The monitoring of arterial stiffness helps provide better understanding of the process and status of atherosclerotic progression in the presence of diabetes [2].

Among several coronary risk factors, physical activity has recently been reported to affect several pathologic conditions including cardiovascular diseases [3]. The increase in physical activity correlates with a decrease in blood pressure and body weight and improvement in endothelial function [4] [5]. In addition, physical activity also improves the state of glucose metabolism [6]. Therefore, the increase in physical activity may have versatile effects on the suppression of atherosclerotic development particularly in the patients with diabetes. However, the exact association between physical activity and the process of atherosclerosis has not been elucidated yet.

In this study, our main aim was to compare the contribution of physical activity to the changes in arterial stiffness between patients with and without diabetes who also have ischemic heart disease.

## Methods

### Subject recruitment

We studied 96 and 109 patients with and without diabetes, respectively, who were diagnosed with ischemic heart disease and treated by percutaneous coronary intervention (PCI). We recruited consecutive patients that were treated by PCI and whose informed consents were obtained. Excluding criteria is unstable clinical condition and significant valvular dysfunction. After PCI, each subject was administered with optimal medical therapy including anti-hypertensive agents and lipid-lowering agents to rigorously control coronary risk factors according to AHA/ACC guidelines [7, 8]. The targets consisted of the following parameters: 1) blood pressure of <140/90 mmHg or 130/80 mmHg in the presence of diabetes; 2) low-density lipoprotein cholesterol <100 mg/dl; and 3) hemoglobin (Hb) A1c <7.0% for diabetes control. The patients were considered to have diabetes if they were undergoing medical treatment with hypoglycemic agents or insulin injections, or if they had HbA1c >6.5% (National Glycohemoglobin Standardization Program) at the time of recruitment. Arterial stiffness was assessed by cardio-ankle vascular index (CAVI) at the time of first diagnosis of significant coronary ischemia and six months after PCI. All sections of a standard informed consent form were explained to each subject, and written informed consent was obtained from all patients. The study protocol conformed to the Declaration of Helsinki and was reviewed and approved by the University of Tokyo Institutional Review Board.(3266)

### CAVI measurements

CAVI was measured by the standardized method using a non-invasive and blood pressure-independent device (VaSera VS-1 500, Fukuda Denshi, Japan) [9]. The examination was performed under fasting conditions and in the room where the standard temperature was maintained. In brief, a cuff was placed on the right and left ankles and the brachial point, electrodes for electrocardiography were attached to both upper arms, and a microphone was placed on the sternal angle for phonocardiography. CAVI measurements were performed in the supine position. Pulse wave velocity (PWV) was calculated by measuring the time between the closing sound of the aortic valve, the notch of brachial pulse wave, and the ankle pulse wave. Using this value, the CAVI value was calculated by the following equation:

$$\begin{array}{l} \text{CAVI}=2\rho /(\text{systolic blood pressure- diastolic blood pressure})\times (\text{ln systolic blood}\\ \text{pressure}/\text{diastolic blood pressure})\times {\text{PWV}}^{2} \end{array}$$

ρ: blood viscosity

We defined the CAVI improved group (Improved-group) as the value of CAVI decrease during follow-up. Others were defined as not changed group (NC-group).

### IPAQ scores

On the other hand, physical activity was assessed using the short form of the International Physical Activity Questionnaire (IPAQ) [10], which estimates physical activity across a comprehensive set of factors to yield a score in metabolic equivalents (METS)-minutes. METs are multiples of resting metabolic rate and multiplying the MET score of an activity by the total number of minutes the activity was performed yields a score of MET-minutes. Vigorous activities such as heavy lifting, digging, speed skiing, running, aerobics, tennis and fast bicycling were counted as 8 METs. On the other hand, moderate activities such as carrying light burdens, swimming slowly, roller skating, doubles tennis were counted as 4 METs. The score was obtained by means of a structured interview conducted by a clinical technologist before administration of PCI.

### Blood collection and analysis

Fasting blood samples were collected for analysis before PCI and six months after PCI. Hemoglobin, platelet count, HbA1c (standardized by the National Glycohemoglobin Standardization Program), estimated glomerular filtration rate (eGFR), total cholesterol, triglycerides, calcium, phosphorus, and brain natriuretic peptide (BNP) were measured using standard laboratory methods at the University of Tokyo hospital.

### Statistics

All values are expressed as mean ± standard deviation. To improve skewed data and kurtosis, variables such as serum levels of BNP and C-reactive protein were log-transformed when possible and then back-transformed to their natural units for presentation. Differences between groups were calculated using the Mann-Whitney U test, t-test, and χ2 test. The potential relationships between parameters were explored using Pearson correlation tests. A p value of <0.05 was considered to be statistically significant. The results of comparisons are represented as box plots (middle dash in the box indicates the median; 25^th^ and 75^th^ percentiles are represented by end caps of the box; whiskers extend to the last observed value). The association between the improvement of CAVI and clinical parameters was assessed by univariate and multivariate regression analyses. Data analyses were performed using the software programs PASW Statistics 18 (SPSS Inc., Chicago, USA) and JMP Pro 9 (SAS Institute, NC, USA). Data is presented in S1 Dataset.

## Results

### Patient characteristics

There were no significant differences in clinical parameters other than the Brinkman index and the value of CAVI between patients with and without diabetes (Table 1). The CAVI values increased in patients with diabetes relative to non-diabetics (9.46 ± 1.09 vs 9.14 ± 1.03, respectively, p = 0.030). According to physical activity parameters, the IPAQ score was not significantly different between patients with and without diabetes. In addition, all medications such as angiotensin II receptor blockers (ARBs), angiotensin converting enzyme inhibitors (ACEIs), and statins were equally prescribed in patients with and without diabetes.

**Table 1:** Patient characteristics.

	Subjects without diabetes	Subjects with diabetes	
N (male / female)	109 (85 /24)	96 (80 / 16)	
Age (years)	67.6 ± 9.5	68.5 ± 8.4	NS
Height (cm)	162.6 ± 9.2	163.8 ± 8.2	NS
Body weight (kg)	65.0 ±13.6	67.0 ± 12.6	NS
Body mass index (kg/m^2^)	24.3 ± 3.6	24.9 ± 3.9	NS
Systolic BP (mmHg)	143.7 ± 21.0	147.3 ± 21.3	NS
Diastolic BP (mmHg)	87.2 ± 10.4	86.8 ± 11.9	NS
Tchol (mg/dL)	172.1 ± 29.3	169.7 ± 38.7	NS
eGFR (ml/min/1.73m^2^)	72.0 ± 17.0	70.1 ± 22.4	NS
uric acid (mg/dL)	5.8 ± 1.3	5.5 ± 1.4	NS
CRP (mg/dL)	0.47 ± 1.43	0.25 ± 0.60	NS
BNP (pg/ml)	53.2 ± 69.8	50.9 ± 51.7	NS
IPAQ score	1545.4 ± 1359.5	1231.2 ± 1087.0	NS
**Brinkman Index**	**508.5 ± 572.4**	**761.8 ± 810.0**	**p < 0.05**
Hypertension	70 (64.2%)	66 (68.8%)	NS
Dyslipidemia	80 (73.4%)	78 (81.3%)	NS
Statin	76 (69.7%)	67 (69.8%)	NS
Beta blocker	18 (16.5%)	13 (13.5%)	NS
ACE I	25 (26.0%)	19 (19.8%)	NS
ARB	36 (33.0%)	37 (38.5%)	NS
Ca blocker	53 (48.6%)	42 (43.7%)	NS
Statin addition	21 (19.2%)	20 (20.8%)	NS
ACEI/ARB addition	24 (22.0%)	20 (20.8%)	NS
Uric acid lowering agents	13 (11.9%)	11 (11.5%)	NS

BP; blood pressure, T chol; total cholesterol, eGFR; estimated glomerular filtration rate, CRP; C-reactive protein, BNP; brain natriuretic peptide, IPAQ; International Physical Activity Questionnaire, ACEI; angiotensin converting enzyme inhibitor, ARB; angiotensin receptor blocker, Ca blocker; Calcium channel blocker, NS; not significant

### Change of CAVI after PCI and optimal medical therapy

During follow-up, CAVI values did not change significantly in patients with and without diabetes. Next we investigated the determining factor CAVI improvement. We defined Improved-group as the value of CAVI decrease during follow-up. Others were defined as NC-group. During follow-up of optimal medical therapy, the CAVI of 48 patients did not change (NC-group), however it did improve for 48 patients (Improved-group) in the diabetes group. By contrast, the CAVI of 59 patients did not change (NC-group), but did improve for 50 patients (Improved-group) without diabetes. With regard to medications, statin was newly prescribed in 41 patients, whereas ACEIs or ARBs were newly added in 44 patients. The addition of these medications did not affect CAVI improvement. (patients with newly prescribed statins vs. patients without it: −0.073 ± 0.86 vs. −0.072 ± 0.91, respectively, p = 0.49) (patients with newly prescribed ARBs or ACEIs vs. patients without it: −0.086 ± 0.87 vs. −0.019 ± 0.99, p = 0.33).

### Determinants of CAVI changes

**Fig 1:**
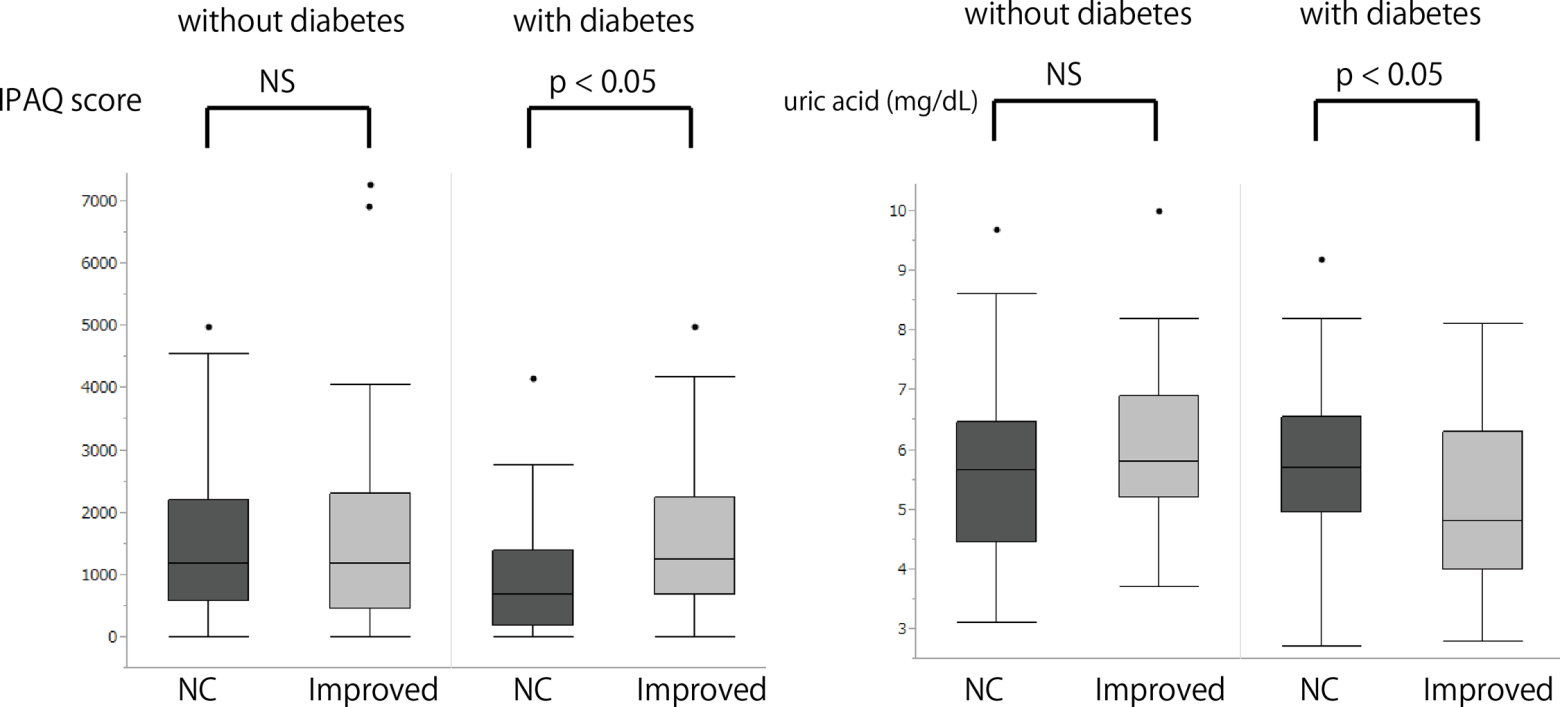
The comparison of IPAQ score and uric acid level between Improved-group and NC-group in patients with and without diabetes.

We next investigated the difference between NC- and Improved-groups in patients with and without diabetes. It revealed that IPAQ scores were significantly higher in the Improved-group in patients with diabetes (937.9 ± 923.2 vs. 1524.6 ± 1166.2, respectively, p < 0.05), while there was no difference in IPAQ scores between these two groups in patients without diabetes (Fig 1). In addition, the level of uric acid was also different between NC-group and Improved-group only in patients with diabetes (5.78 ± 1.27 vs. 5.13 ± 1.47, respectively, p < 0.05).

**Fig 2:**
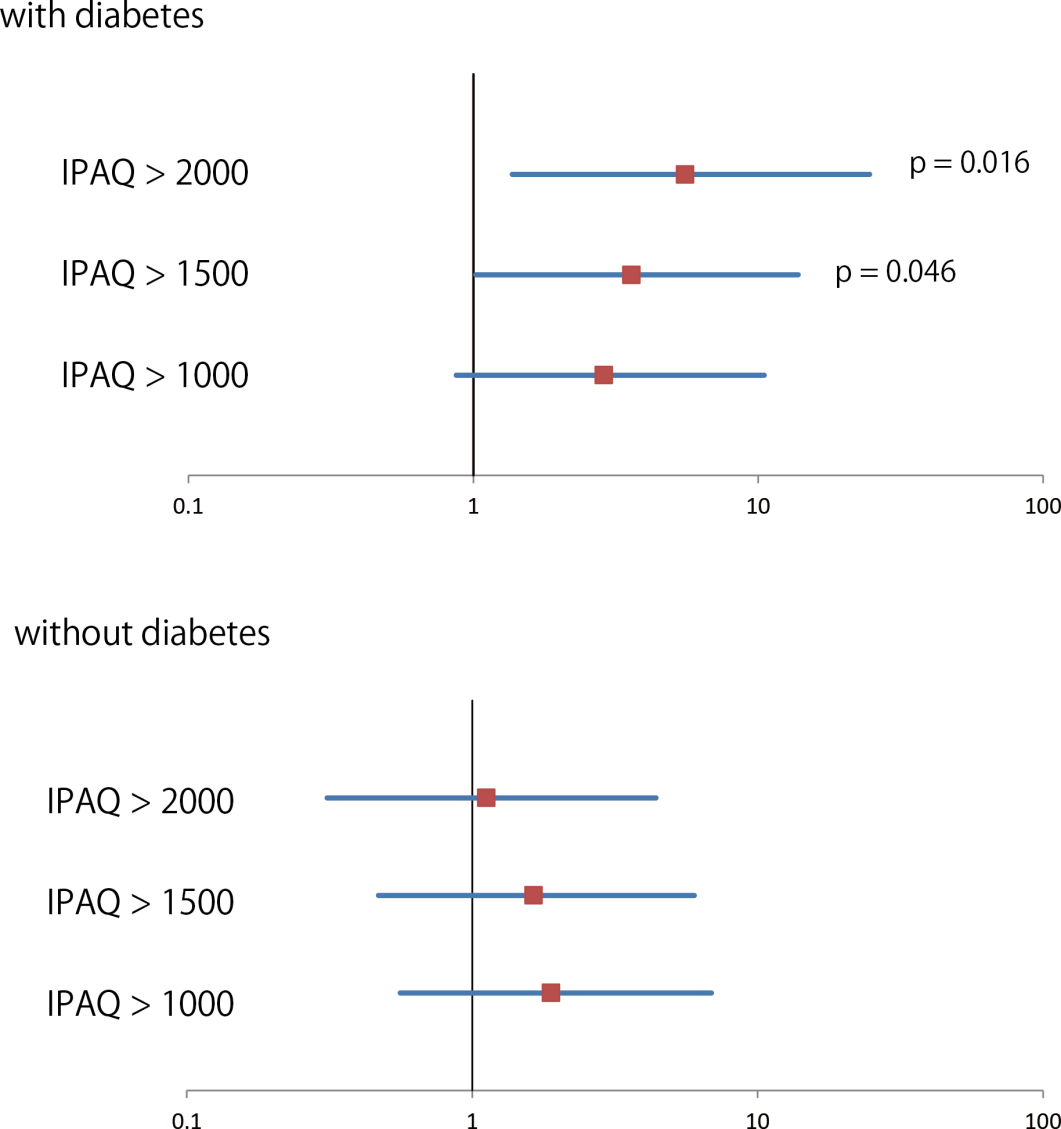
Odds ratio and 95% confidence interval of the improvement of CAVI during follow-up in patients with and without diabetes. Model adjusted for sex, age, blood pressure, the value of CAVI in the baseline, total cholesterol, eGFR, and uric acid.

Indeed, the different factors between non-diabetics and diabetics correlated with CAVI improvement. Univariate regression analyses demonstrated that age, value of baseline CAVI, total cholesterol, and eGFR correlated with CAVI improvement in patients without diabetes, whereas uric acid and IPAQ scores significantly affected the improvement of CAVI in patients with diabetes (Table 2, Fig 1, S1 Fig). Multivariate regression analyses, including age, sex, value of CAVI baseline, total cholesterol, eGFR, uric acid, and IPAQ scores, revealed that CAVI baseline and age were independently related to CAVI changes in patients without diabetes, whereas uric acid and IPAQ scores both affected patients with diabetes. Subgroup analyses demonstrated that the setting threshold of IPAQ >1500 appeared to correlate with CAVI improvement in patients with diabetes after adjusting sex, age, blood pressure, the value of CAVI in the baseline, total cholesterol, eGFR, and uric acid (Fig 2). Among clinical parameters including blood pressure, there were no parameters that changed significantly during follow up in each group of NC-group and Improved-group.

**Table 2:** Odds ratio (p-value) of the improvement of CAVI during follow-up.

	Without diabetes		With diabetes	
	Univariate	Multivariate	Univariate	Multivariate
Age	0.93 (0.0041)	0.90 (0.019)	1.008 (0.73)	
Sex	0.52 (0.17)		1.36 (0.58)	
Systolic BP	1.005 (0.53)		1.00 (0.79)	
Diastolic BP	1.03 (0.12)		1.01 (0.52)	
CAVI (baseline)	1.54 (0.032)	3.20 (0.0009)	1.46 (0.068)	
Tchol	1.009 (0.21)		0.99 (0.53)	
eGFR	1.03 (0.049)		1.02 (0.14)	
Uric acid	1.34 (0.078)		0.71 (0.031)	0.62 (0.022)
IPAQ	1.00 (0.66)		1.0005 (0.011)	1.0007 (0.0085)

BP; blood pressure, CAVI; cardio-ankle vascular index, T chol; total cholesterol, eGFR; estimated glomerular filtration rate, IPAQ; International Physical Activity Questionnaire

## Discussion

### Change of arterial stiffness

The parameters concerning vascular stiffness such as PWV and CAVI are good predictive marker of atherosclerotic disease [11]. The relationship between these parameter values and clinical variables has been reported, whereas there is limited information on determinants of the change in aortic stiffness. Tomiyama et al. demonstrated that both raised blood pressure and plasma glucose significantly accelerated the progression of arterial stiffness. Matsumae et al. demonstrated that the clinical factors that accelerate aortic stiffening occurred in patients undergoing hemodialysis. They found that age, HDL, LDL, and HCV infection were associated with annual worsening of PWV [11]. Jung Y et al. reported that mean arterial pressure and triglyceride levels were factors affecting the change of PWV in patients undergoing peritoneal dialysis [12]. These previous studies did not include physical activity factors. In addition, these studies focused on the determining factors that deteriorate vascular function, whereas the current study focused on factors that have an impact on the improvement in arterial stiffness after optimal medical therapy, such as medications of hypertension and hyperlipidemia.

### Pharmacological effect on the change of arterial stiffness

According to the pharmacological interventions directed at changing the CAVI levels, several therapeutic interventions were reported to improve arterial stiffness. A six-month course of ACE inhibitors with perindopril produced no significant changes in blood pressure, but a significant decrease in PWV occurred, suggesting diminished arterial wall stiffness [13]. Matsuo et al. demonstrated that statin therapy improves arterial stiffness via both anti-oxidant and -inflammatory properties [14]. These previous reports suggested that six months was sufficient duration to investigate the change of arterial stiffness by various interventions. However, in the current study, these optimal medical therapies such as statin medication were administered equivalently to each group, so that no therapeutic parameters significantly affected the improvement in arterial stiffness between patients with and without diabetes. Therefore, the differences in determining the factors that affect the CAVI improvement between patients with and without diabetes are supposed to be derived from the intrinsic mechanism. Indeed, significantly higher CAVI scores were observed in patients with type 2 diabetes compared with non-diabetic patients [15, 16] and may have a tremendous impact on the behavior of the progression in arterial stiffness. In addition, the current study includes patients with much higher coronary risks, which may alter the determining factors that affect arterial stiffness observed in other studies. As a result, the newly added statin or ARBs or ACEIs did not contribute to the CAVI improvement.

### Physical activity

In contrast, physical activity was recently reported to affect the arterial stiffness in patients with atherosclerosis, leading to lowering coronary risk [15, 17]. Funck et al. demonstrated that low physical activity corresponded to increased arterial stiffness in type 2 diabetics [16]. The beneficial effect of increased physical activity has been suggested to occur in a specific manner: Exercise-induced enhancement of blood flow leads to the augmentation of shear stress. This shear stress stimulates endothelial derived nitric oxide production and this induces arterial structural adaptations [18].

In addition to these direct effects on vasculature, physical activity was reported to improve the status of diabetes [19]. There is plenty of evidence for the beneficial effects of physical activity on glycemic control, and it can lead to the lowering of cardiovascular mortality [20, 21]. Its mechanism may relate to the decrease of systemic low-grade inflammation due to exercise in type 2 diabetics [22]. The results in the current study indicate that the effect of physical activity on arterial stiffness was greater in patients with diabetes; this seems to be derived from multiple factors that are affected by increased physical activity. The effect of increased physical activity is supposed to be amplified by the multiple pathways. Therefore, the maintenance of physical activity become a more important issue in the high risk patients and should be introduced in high risk patients more rigorously.

However the raw value of CAVI had no association with the value of IPAQ score (S2 Fig). From this finding, physical activity did not affect the value of arterial stiffness directly, and the level of physical activity was considered to correlate the reactivity of optimal medical therapy after PCI.

### Uric acid

Another finding in this study is the relationship between arterial stiffness and serum uric acid level. The level of uric acid was significantly lower in the Improved-group of patients with diabetes, whereas uric acid lowering agents were similarly prescribed (NC: 8 (16.7%), Improved: 3 (6.3%)). Some previous papers have demonstrated similar results, however the exact mechanism underlying these results remain unclear [23, 24]. Some possible mechanisms have been proposed, including high uric acid effects on the increase of the proliferation of vascular smooth muscle cells and the impairment of nitric oxide generation [25]. However, the reasons why the close relationship between CAVI change and uric acid were observed only in the patients with diabetes have not been clarified in the current study.

## Conclusion

The determining factors that affect CAVI improvements during follow-up were significantly different between patients with and without diabetes. IPAQ scores and uric acid levels were significantly correlated with CABI changes.

## Study Limitations

The study cohort consisted of adult patients referred for PCI, who may differ from the general population or subjects with ischemic heart disease in terms of the prevalence of underlying medical conditions. However, the patients represented individuals at high atherosclerotic risk, and the results could apply to other subject groups with similar atherosclerotic risks. We checked IPAQ scores only once before PCI and we could not follow the change of physical activity in the current study.

This study was limited by its retrospective design; therefore, we cannot make any conclusions with regard to causality between physical activity levels and arterial stiffness. In addition, the inconsistency of uric acid lowering agent prescriptions complicated the problem of the effects of these agents in treating arterial stiffness; the design of the current study was limited with regard to determination of these agents' effects on the arterial stiffness improvement. The sample size of this study was not sufficiently large, so future studies with a larger number of patients are needed to investigate the improvement of arterial stiffness in patients with atherosclerotic diseases. There may be an effect derived from the phenomenon of the “regression to the mean (RTM)”, i.e., higher values of CAVI in baseline tend to decrease and lower values tend to increase on a second measurement [26]. Indeed, univariate regression analysis revealed that baseline CAVI affected the improvement of CAVI in patients with and without diabetes. However, in patients with diabetes there was no significant difference of baseline CAVI between patients with Improved-group and NC-group (9.25 ± 1.07 vs 9.67 ± 1.07, p = 0.062). The significant association between baseline CAVI and the improvement of CAVI was not identified in patients with diabetes after multivariate regression analyses. Therefore RTM effect was considered to be comparatively small in patients with diabetes.

## Supporting Information

S1 Dataset Dataset in the current study. (XLSX)

S1 Fig The relationship between the change of CAVI and IPAQ score in patients with diabetes. There was a significant negative correlation between these two.
(PPTX)

S2 Fig The relationship between raw value of CAVI in baseline and IPAQ score. There were no associations between raw value of CAVI in baseline and IPAQ score in subjects with and without diabetes.
(PPTX)
