# Supplementary material for: Different Contributions of Physical Activity on Arterial Stiffness between Diabetics and Non-Diabetics
Source: PLoS One. 2016 Aug 10;11(8):e0160632. doi: 10.1371/journal.pone.0160632 (PMC4980026; doi:10.1371/journal.pone.0160632)
Supplement: S1 Fig — There was a significant negative correlation between these two. (PPTX) [file pone.0160632.s002.pptx]

## Slide 1
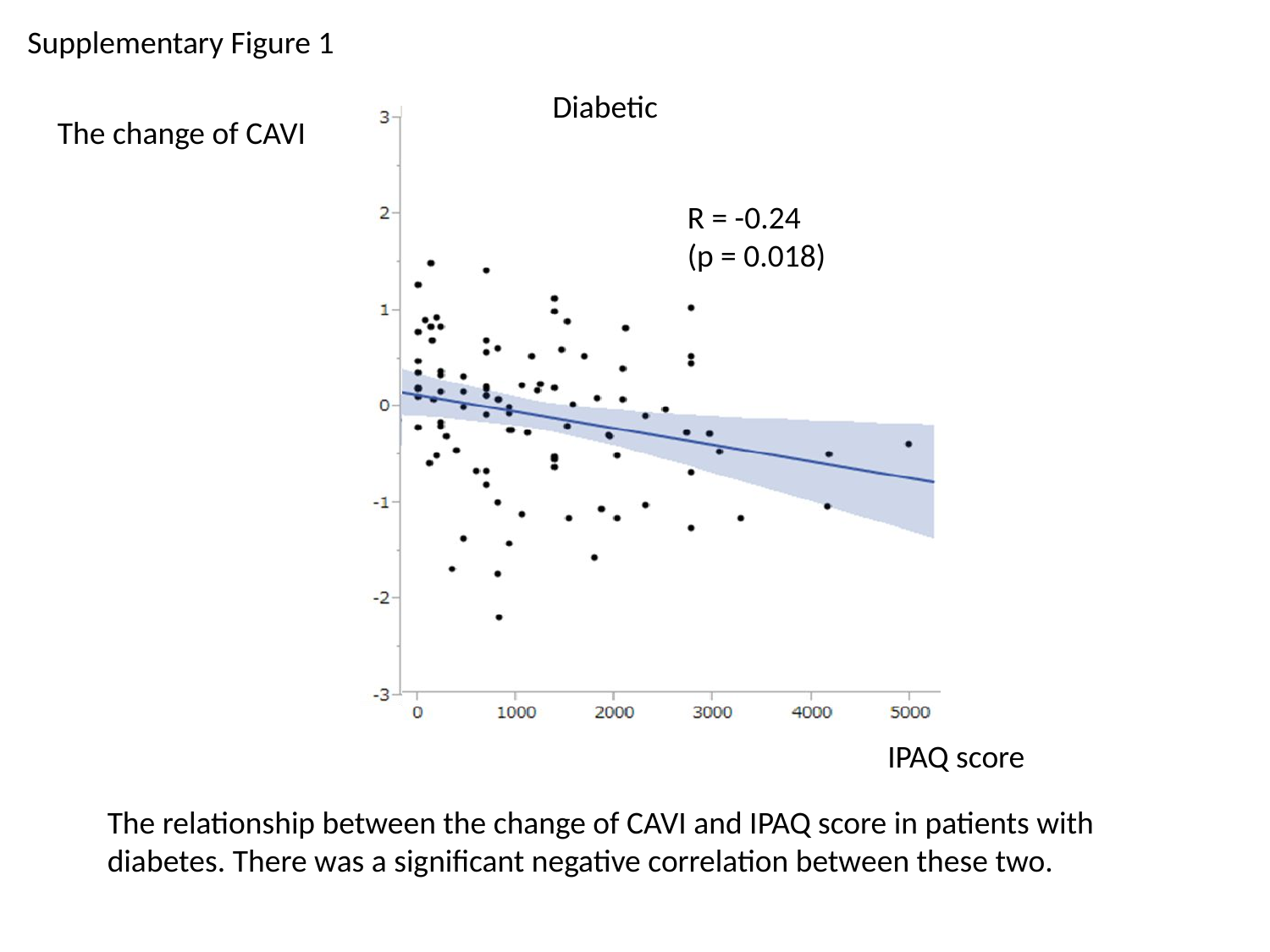

Supplementary Figure 1
Diabetic
The change of CAVI
R = -0.24
(p = 0.018)
IPAQ score
The relationship between the change of CAVI and IPAQ score in patients with diabetes. There was a significant negative correlation between these two.
