# Supplementary material for: Different Contributions of Physical Activity on Arterial Stiffness between Diabetics and Non-Diabetics
Source: PLoS One. 2016 Aug 10;11(8):e0160632. doi: 10.1371/journal.pone.0160632 (PMC4980026; doi:10.1371/journal.pone.0160632)
Supplement: S2 Fig — There were no associations between raw value of CAVI in baseline and IPAQ score in subjects with and without diabetes. (PPTX) [file pone.0160632.s003.pptx]

## Slide 1
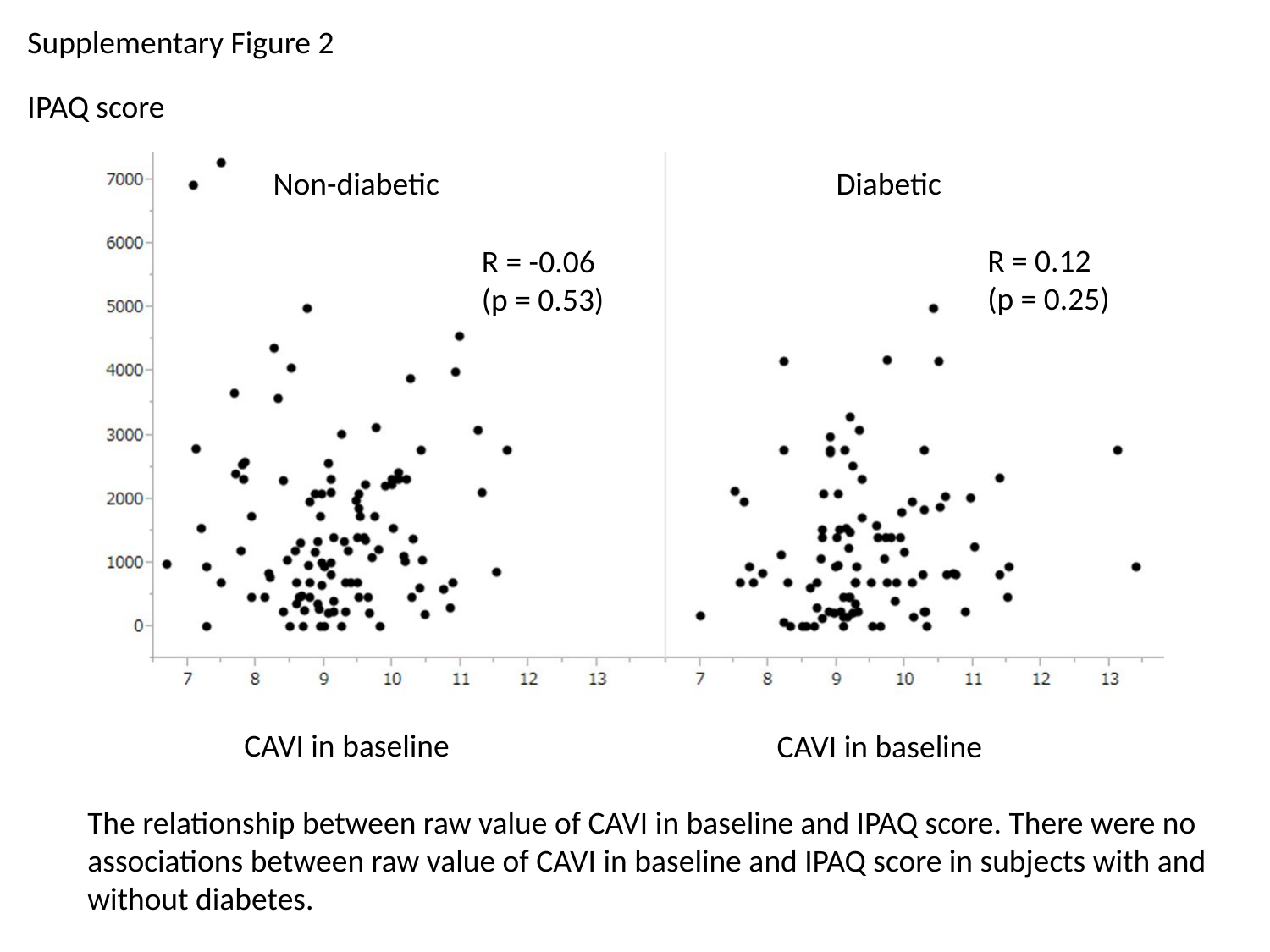

Supplementary Figure 2
IPAQ score
Non-diabetic
Diabetic
R = 0.12
(p = 0.25)
R = -0.06
(p = 0.53)
CAVI in baseline
CAVI in baseline
The relationship between raw value of CAVI in baseline and IPAQ score. There were no associations between raw value of CAVI in baseline and IPAQ score in subjects with and without diabetes.
